# Carbapenemase-encoding genes in critical gram-negative bacteria isolated from ICU patients with infections and/or gastrointestinal carriage, and environmental samples in the amhara National Regional state, Ethiopia

**DOI:** 10.1371/journal.pone.0330613

**Published:** 2025-09-04

**Authors:** Mizan Kindu, Feleke Moges, Ashenafi Alemu, Dawit Hailu, Rafael Joseph, Esmael Besufikad, Dareskedar Tsehay, Daniel Bashah, Zemene Tigabu, Adane Mihret, Baye Gelaw

**Affiliations:** 1 Department of Medical Microbiology, University of Gondar, Gondar, Ethiopia; 2 Department of Medical Laboratory Science, School of Medicine, MaddaWalabu University, Bale Goba, Ethiopia; 3 Armauer Hansen Research Institute, Addis Ababa, Ethiopia; 4 Department of Medical Laboratory Science, College of Health Sciences, Addis Ababa University, Addis Ababa, Ethiopia; 5 Department of Pediatrics and Child health, University of Gondar, Gondar, Ethiopia; 6 Department of Microbiology, Immunology and Parasitology, School of Medicine, College of Health Sciences, Addis Ababa University, Addis Ababa, Ethiopia; Yamagata University Faculty of Medicine: Yamagata Daigaku Igakubu Daigakuin Igakukei Kenkyuka, JAPAN

## Abstract

Effective infection control requires identifying and eliminating carbapenemase-producing (CP) Gram-negative bacteria (GNB) in high-risk groups like intensive care unit (ICU) patients and from contaminated environmental surfaces. This study aimed to describe the diversity of carbapenemase-encoding genes among critical GNB isolates from ICU patients with infection and/or gastrointestinal (GI) colonization, as well as from ICU environmental surfaces in the Amhara National Regional state, Ethiopia.A total of 169 carbapenem-resistant isolates were identified, including 26 from infections, 82 from GI colonization, and 61 from environmental samples. These comprised *Klebsiellapneumoniae* (n = 107), *Escherichia coli* (n = 16), *Pseudomonas aeruginosa* (n = 9), and *Acinetobacter* species (n = 37), of which 147 were analyzed.Singleplex and multiplex PCR were performed to detect predominant carbapenemase-encoding genes, including KPC (*bla*_KPC_), MBLs (*bla*_IMP_, *bla*_VIM_, and *bla*_NDM_), and OXA (*bla*_OXA-48_, *bla*_OXA-23_, and *bla*_OXA-58_). PCR analysis revealed that at least one carbapenemase-encoding gene was detected in 133 (78.7%) of the 169 carbapenem-resistant isolates from patients and environmental surfaces. They were detected in 22/133(16.5%), 70/133(52.6%), and 41/133(30.8%) isolates obtained from infection, GI colonization, and environmental samples, respectively. The most prevalent carbapenemase-encoding gene, *bla*_NDM_, was found in 101 (75.9%) of the 133 CP isolates. Other detected carbapenemase-encoding genes included *bla*_OXA-23_ in 5 (3.8%) isolates, *bla*_VIM_ in 4 (3.0%), *bla*_OXA-48_ in 1 (0.8%), and *bla*_OXA-58_ in 1 (0.8%). *K. pneumoniae* and *Acinetobacter* spp. harbored all the gene types detected in this study. Co-harboring of two or more carbapenemase-encoding genes, in combination with *bla*_NDM_, was observed in 21 of 133 (15.8%) isolates, including 7 *K. pneumoniae* and 14 *Acinetobacter* spp. isolates. However, *bla*_KPC_ and *bla*_IMP_ were not identified in any of the tested isolates. This study highlights the presence of carbapenemase-encoding genes among critical GNB across all sample source types in the study area. Moreover, the detection of isolates harboring multiple carbapenemase-encoding genes underscores the need for enhanced infection control measures in ICU settings.

## Introduction

Carbapenems have become the last resort antibiotics for treating multidrug-resistant (MDR) Gram negative bacterial (GNB) infections, particularly in hospital settings [1]. However, the emergence of carbapenem resistant bacteria is threatening the effectiveness of these antibiotics. Carbapenemase enzymes are the most common resistance mechanism to carbapenem worldwide [2]. Initially, carbapenemases were chromosomally mediated in a few specific species, but now they are mediated by a plasmid, or both a chromosomal and a plasmid. This results in a more violent spread of resistance due to clonal expansion and horizontal gene transfer between different bacterial species and genera [3,4]. Carbapenem resistance has been labeled in Enterobacterales, mostly in *Klebsiella pneumoniae* compared to *Escherichia coli* or other Enterobacterial species, and in non-fermentativeGram-negative bacilli such as *Acinetobacterbaumannii*and *Pseudomonas aeruginosa*. Moreover, World Health Organization (WHO) listed these carbapenem-resistant GNB isolates as pathogens of critical importance [5].

Carbapenemases can be classified into various categories according to the Ambler classification scheme as A, B, and D. Globally, the most common carbapenemases consist of *K*.*pneumoniae*carbapenemases (KPC) are clinically the most common enzymes among the class A carbapenemases. New Delhi metallo-β-lactamases (NDM), Verona integronencodedmetallo-β-lactamases (VIM), and imipenemase (IMP) types belong to class B metallo-β-lactamases (MBLs), whereas oxacillinases, including OXA-23, OXA-24, OXA-58, and OXA-48, are part of the class D enzymes [2]. A few studies conducted in Ethiopia have provided evidence of infections caused by carbapenemase-producing (CP) GNB and have reported NDM carbapenemase being the most commonly identified enzyme [6,7].

For effective infection control, it is crucial to identify and eliminate carbapenemase-producing critical GNB among high-risk groups, such as intensive care unit (ICU) patients, and contaminated environmental sources [8]. Additionally, gastrointestinal(GI) colonized patients serve as reservoirs for CP pathogens, facilitating their dissemination [9]. Therefore, this study aimed to describe the diversity of genes encoding carbapenemase enzymes among critical GNB isolates from ICU patients with GI colonization and/or infection, as well as from ICU environmental samples in the AmharaNational Regional state, Ethiopia.

## Materials and methods

### Study area, patients, and specimen collection

Samples were obtained from patients and environmental surfaces in the ICUs of the University of Gondar Comprehensive Specialized Hospital (UoGCSH) and FelegeHiwot Comprehensive Specialized Hospital (FHCSH), located in the AmharaNational Regional state, North-West Ethiopia, the 1^st^ of June 2021 to the 30^th^, December 2022.During the study period, non-duplicated clinical samples were collected from each of the 600 study participants based on clinical suspicion of infection. These included blood, urine, pus/wound swabs, and other specimens. In addition to the clinical samples, rectal swabs were collected from these study participants to identify GI colonization with CP critical GNB. Simultaneously, 384 environmental swab samples were collected from ICUs during the same period [10].

### Critical GNB isolates with reduced susceptibility to carbapenem

Bacterial identification using various biochemical tests, antibiotic susceptibility testing by disk diffusion technique, and phenotypic detection of carbapenemase enzymes were performed as described in our previously published work by Mizan et al. [10]. A total of 169 carbapenem-resistant isolates were identified, including 26 from infections, 82 from gastrointestinal colonization, and 61 from environmental samples. These comprised *K. pneumoniae* (n = 107), *E. coli* (n = 16), *P. aeruginosa* (n = 9), and *Acinetobacter* spp. (n = 37) (S1 Table).

From carbapenem-resistant critical GNB isolates, rectal and environmental isolates that tested phenotypically positive for carbapenemase production using the mCIM test were subjected to carbapenemase-encoding gene detection in the case of *K. pneumoniae*, *E. coli*, and *P. aeruginosa*. However, for clinical samples and *Acinetobacter* spp., all carbapenem-resistant isolates identified by the disk diffusion test were included for carbapenemase-encoding gene detection using conventional PCR [11].Therefore, total of 147 these isolates were stored at –20 °C, and transported to the Armauer Hansen Research Institute (AHRI) for PCR analysis.

### DNA extraction

DNA was extracted from carbapenem-resistant critical GNB isolates using the boiling method, as previously described [12]. Briefly, each isolate was grown overnight on nutrient agar (Oxoid, UK), and 3 to 5 colonies were suspended in 300 µL of 1 × Tris-EDTA buffer. The suspension was subjected to boiling at 94°C for 10 minutes in a water bath (Thermo Fisher Scientific, CA, USA), followed by freezing at −20°C for 10 minutes, incubation at room temperature for 1 minute, and centrifugation at 14,000 *× g* for 5 minutes. Finally, 150 µL of the supernatant was transferred into a nuclease-free Eppendorf tube and assessed using a Nanodrop (Thermo Scientific, USA) to measure the quality and quantity of the extracted DNA. The samples were then stored at −20°C until further analysis.

### PCR-based detection of carbapenemase genes

*K*. *pneumoniae*, *E*. *coli*, *P*.*aerugniosa* and *Acinetobacter* spp.isolates resistant to carbapenems were analyzed for the presence of carbapenemase-encoding genes using PCR-based methods. Singleplex and multiplex PCR were performed to detect the most common carbapenemase-encoding genes, including KPC (*bla*_KPC_), MBLs (*bla*_IMP_, *bla*_VIM_, and *bla*_NDM_), and OXA (*bla*_OXA-48_, *bla*_OXA-23_, and *bla*_OXA-58_), using a panel of primers as previously described [11].The sets of specific primers used for the detection of carbapenemase genes are shown in supplementary table (S2 Table).

For both singleplex and multiplex PCR, the reaction conditions were optimized by testing a range of annealing temperatures to determine the optimal annealing conditions for all primers. PCR was performed in a total volume of 25 µL, consisting of 12.5 µL (final concentration 1×) of HotStarTaq Master Mix (Qiagen, Hilden, Germany), 5 µL of genomic DNA, 0.4 µM of each primer, and 5.5 µL of nuclease-free water.

PCR amplification of carbapenemase-encoding genes was carried out under the following cycling conditions: initial denaturation at 95°C for 5 seconds, followed by 35 cycles of denaturation at 94°C for 30 seconds, annealing at 57°C for 1 minute, and extension at 72°C for 1 minute, with a final extension at 72°C for 5 minutes. The PCR amplicons were analyzed by 1.5% agarose gel electrophoresis stained with ethidium bromide. The presence or absence of carbapenemase genes was determined using a UV transilluminator (GelDoc, Bio-Rad), and PCR product bands were compared against a 100 bp DNA ladder.

### Quality control

For the optimization of the multiplex PCR, known control strains were used as positive controls. DNA samples from *K. pneumoniae* ATCC 1706 served as a negative control for *bla*_KPC_, *bla*_NDM_, *bla*_OXA-48_, and *bla*_VIM_. *K. pneumoniae* ATCC BAA-1705, *K. pneumoniae* ATCC BAA-2146, *P. aeruginosa* AR Bank #00546, and *K. pneumoniae* AR Bank #0039 were used as positive controls for *bla*_KPC_, *bla*_NDM_, *bla*_VIM_, and *bla*_OXA-48_, respectively, all in conjunction with carbapenemase detection. Before multiplexing the primers for carbapenemase-encoding genes, each primer was tested individually using a singleplex PCR reaction. The DNA samples for both negative and positive controls were obtained from the Ethiopian Public Health Institute

### Ethical approval and consent/assent to participate

Ethical approval was obtained from the institutional review board of the University of Gondar with reference number O/VIP/RCS/05/1773/2019. Informed written consent was obtained from each study participant with the help of caregivers. Briefly, since most of the study participants were neonates, assent was taken from their parents or guardians. Moreover, consent was obtained from the caregiver for critically ill or unconscious individuals whose age was 18 years and above.

## Results

A total of 169 carbapenem resistance isolates of critical GNB were recovered phenotypically. CP critical GNB isolates carrying carbapenemase-encoding genes were identified across all sample source, obtained from ICU patients with an infection and/or GI colonization, as well as from environmental surfaces (Table 1). Carbapenemase-encoding genes were detected in 133 (78.7%) of the 169 carbapenem resistance isolates. Among CP critical GNB, the majority were *K*. *pneumoniae* 87 (65.4%), followed by *Acinetobacter*spp. 31 (23.3%), *E*. *coli* 12 (9%) and *P*. *aeruginosa* 3 (2.3%).

**Table 1:** PCR confirmed carbapenemase positive critical GNB obtained from ICU patients with GI colonization and/orinfection, as well as from ICU environmental samples in the AmharaNational Regional state, Ethiopia, 2021 to 2022.

Bacterial species	Sample source			
	Infection N (%)	GI Colonization N (%)	Environmental N (%)	Total N (%)
*K*. *pneumoniae*	12 (13.8)	54(62.1)	21(24.1)	87 (100)
*E*. *coli*	0(0)	10 (83.3)	2 (16.7%)	12 (100)
Acinetobacter spp.	9 (29.0)	6 (19.4)	16 (51.6)	31 (100)
*P*. *aeruginosa*	1 (33.3)	0(0)	2 (66.7)	3(100
Total	22 (16.5)	70 (52.6)	41(30.8)	133(100)

### Characteristics of PCR confirmed carbapenemase positive ICU patients

Critical GNB carrying carbapenemase-encoding genes were detected among ICU patients with prevalence of an infection and GI colonization rate of 3.7% (22/600) and 11.3% (68/600), respectively. Five patients had both an infection and GI colonization, while two other patients had GI colonization with mixed species of CP critical GNB.Majority of the patients were from the neonatal ICU (NICU) (n = 62) (Table 2).

**Table 2:** Characteristics of PCR confirmed carbapenemase positive ICU patientswith GI colonization and/or infection in the AmharaNational Regional state, Ethiopia, 2021 to 2022.

		Infection(n)	GI Colonization(n)	Both infection and GI colonization(n)	Total(n)
ICU	NICU	11	47	4	62
	Other ICU	6	16	1	23
Sex	Male	7	34	2	43
	Female	10	29	3	42
Sources of isolates	Blood	14	0	5	19
	Urine	2	0	0	2
	Discharge	1	0	0	1
	Rectal swab	0	63	0	63
Patients referral	Yes	7	26	3	36
	No	10	37	2	49
Invasive procedure	Yes	16	61	5	82
	No	1	2	0	3
Empirical antibiotic therapy	Yes	16	56	5	77
	No	1	7	0	8

### Distribution of carbapenemase encoding genes detected among critical Gram negative bacteria

The most prevalent carbapenemase-encoding gene detected was *bla*_NDM_, 101/133 (75.9%) followed by *bla*_OXA-23_ gene, 5/133 (3.8%); *bla*_VIM_ 4/133 (3.0%); *bla*_OXA48_ 1/133(0.8%) and *bla*_OXA-58_ gene 1/133(0.8%) isolates. Co-harboring of two or more types of carbapenemase-encoding genes with *bla*_NDM_ genes were observed in 21/133(15.8%) isolates. *bla*_NDM +_ *bla*_OXA-23_ were detected in 9 isolates; *bla*_NDM_ + *bla*_VIM_ in 5 isolates; *bla*_NDM_ + *bla*_OXA-58_ in 4 isolates; *bla*_NDM_ + *bla*_OXA-48_ in 1 isolate; *bla*_NDM_ + *bla*_OXA-23 _+ *bla*_VIM_ in 1 isolate and *bla*_NDM_ + *bla*_OXA-23_ + *bla*_OXA-48_ in 1 isolate. However, *bla*_KPC_, and *bla*_IMP_ genes were not detected (S1 Fig).

**Fig 1:**
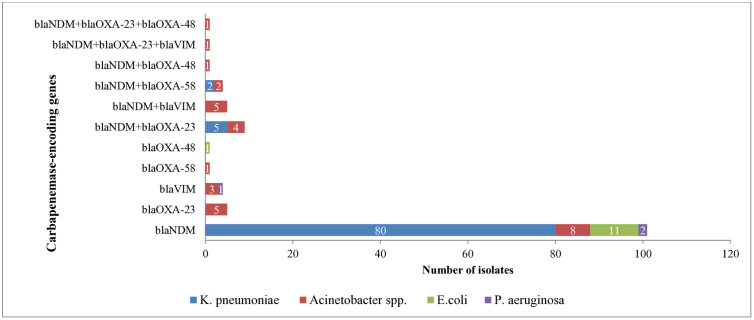
Distribution of carbapenemase-encoding genes among critical GNB obtained from ICU patients with GI colonization and/orinfection, as well as from ICU environmental samples in the AmharaNational Regional state, Ethiopia, 2021 to 2022.

The *bla*_NDM_ gene was identified across all species, including 87 *K. pneumoniae*, 22 *Acinetobacter*spp., 11 *E. coli*, and 2 *P. aeruginosa*. The *bla*_OXA-23_ gene was found in 5 *Acinetobacter*spp., while *bla*_VIM_ was detected in 3 *Acinetobacter*spp. and 1 *P. aeruginosa*. The *bla*_OXA-58_ gene was identified in 1 *Acinetobacter*spp., and the *bla*_OXA-48_ gene was identified in 1 *E. coli* isolate. Co-harboring of two or more carbapenemase-encoding genes, in combination with *bla*_NDM_, was observed in 7 *K. pneumoniae* and 14 *Acinetobacter*spp. (Fig 1).

### Distribution of critical GNB carrying carbapenemase-encoding genes in different ICU environmental surfaces

Critical GNB carrying carbapenemase-encoding genes were detected on various ICU environmental surfaces, with *K. pneumoniae* and *Acinetobacter* spp. being the most frequently isolated species. *K. pneumoniae* carrying *bla*_NDM_ carbapenemase was primarily detected on baby incubators (n = 7), baby bed sets (n = 5), and over-bed tables (n = 3). *Acinetobacter* spp. exhibited a wider range of carbapenemase-encoding genes, including *bla*_NDM_, *bla*_VIM_, *bla*_OXA-23_, and *bla*_OXA-58_, with notable contamination on baby bed sets, bed rail surfaces, mattress,and over-bed tables. *P. aeruginosa* carrying *bla*_NDM_ was found on over-bed table (n = 1) and sink (n = 1), while *E. coli* carrying *bla*_NDM_ was detected on the sink (n = 1) and mattress (n = 1) (Table 3).

**Table 3:** Distribution of carbapenemase-encoding genes among critical GNB in different ICU environmental surfaces in the AmharaNational Regional state, Ethiopia, 2021 to 2022.

	*K*. *pneumoniae*	Acinetobacter spp.	*E. coli*	*P. aeruginosa*
Baby incubator	*bla*_NDM_(7)	*bla*_NDM_(1), *bla*_VIM_(1)	ND*	ND*
Baby bed set	*bla*_NDM_(5)	*bla*_NDM_(1), *bla*_OXA-58_(1), *bla*_NDM_ + *bla*_VIM_(1)	ND*	ND*
Mattress	ND*	*bla*_OXA-23_(1)	*bla*_NDM_(1)	ND*
Bed rail surface	*bla*_NDM_(1)	*bla*_NDM_ + bla_VIM_(1),*bla*_NDM_ + *bla*_OXA-23_(1), *bla*_NDM_ + bal_VIM_ + *bla*_OXA-23_(1)	ND*	ND*
Over bed table	*bla*_NDM_(3)	*bla*_OXA-23_(1), *bla*_NDM_ + *bla*_OXA-23_(1)	ND*	*bla*_NDM_(1)
Baby examination table	*bla*_NDM_(1)	ND*	ND*	ND*
Bed sheet	ND*	*bla*_NDM_(1), *bla*_VIM_(1), *bla*_NDM_ + *bla*_OXA-23_(1)	ND*	ND*
IV set	*bla*_NDM_(1) *bla*_NDM_ + *bla*_OXA-58_(1)	ND*	ND*	ND*
Sink	*bla*_NDM_(2)	*bla*_NDM_(1), *bla*_OXA-23_(1)	*bla*_NDM_(1)	*bla*_NDM_(1)

ND*= Not detected.

### Distribution of carbapenemase encoding genes detected among different sample sources

Carbapenemase-encoding genes were distributed across infection, GI colonization, and environmental samples. Among *K. pneumoniae* isolates, *bla*_NDM_ (n = 80) was detected in infection, GI colonization, and environmental samples. Co-harbored gene, *bla*_NDM_* + bla*_OXA-58_ (n = 2) was found in both GI colonization and environmental sources while *bla*_NDM_ + *bla*_OXA-23_ (n = 5) was only detected in GI colonization. In *Acinetobacter* spp., *bla*_NDM_ (n = 8) were present across all sample types, whereas *bla*_OXA-23_ (n = 5) was detected from GI colonization and environmental samples. Additionally, combinations genes such as *bla*_NDM_ + bla_VIM_ (n = 5) and *bla*_NDM_ + bla_OXA*-*23_ (n = 4), were identified both in infection and environmental samples. For *E. coli*, *bla*_NDM_ (n = 11) was detected in GI colonization and environmental, while *bla*_OXA-48_ (n = 1) was limited to GI colonization. Among *P. aeruginosa*, *bla*_NDM_ (n = 2) was found in environmental samples, and *bla*_VIM_ (n = 1) was identified in an infection (Table 4).

**Table 4:** Carbapenemase-encoding genes among critical GNB in different sample sources in the AmharaNational Regional state, Ethiopia, 2021 to 2022.

Isolates	Carbapenemase-encoding genes	Sample source					Total N(%)		
		Infection N(%)	Colonization N(%)		Environment N(%)	
*K*. *pneumoniae*	*bla*_NDM_	12(15)	48(60)		20(25)	80(100)
	*bla*_NDM_ + *bla*_OXA-23_	0(0)	5(100)		0(0)	5(100)
	*bla*_NDM_ + *bla*_OXA-58_	0(0)	1(50)		1(50)	2(100)
*Acinetobacter* spp.	*bla*_NDM_	1(12.5)	3(37.5)		4(50)	8(100)
	*bla*_VIM_	1(33.3)	0(0)		2(66.7)	3(100)
	*bla*_OXA-23_	0(0)	2(40)		3(60)	5(100)
	*bla*_OXA-58_	0(0)	0(0)		1(100)	1(100)
	*bla*_NDM_ + *bla*_VIM_	3(60)	0(0)		2(40)	5(100)
	*bla*_NDM_ + *bla*_OXA-23_	1(25)	0(0)		3(75)	4(100)
	*bla*_NDM_ + *bla*_OXA-58_	2(100)	0(0)		0(0)	2(100)
	*bla*_NDM_ + *bla*_OXA-48_	0(0)	1(100)		0(0)	1(100)
	*bla*_NDM_ + *bla*_OXA-23_ + *bla*_OXA-48_	1(100)	0(0)		0(0)	1(100)
	*bla*_NDM_ + *bla*_OXA-23_ + *bla*_VIM_	0(0)	0(0)		1(100)	1(100)
*E*. *coli*	*bla*_NDM_	0(0)	9(81.8)		2(18.2)	11(100)
	*bla*_OXA-48_	0(0)	1(100)		0(0)	1(100)
*P*.*aerugenosa*	*bla*_NDM_	0(0)	0(0)		2(100)	2(100)
	*bla*_VIM_	1(100)	0(0)		0(0)	1(100)
Total N(%)		22(16.5)	70(52.6)		41(30.8)	133(100)

## Discussion

This study detected carbapenemase-encoding genes in CP critical GNB isolated from ICU environmental surface contamination, GI colonization, and infections in ICU patients, using environmental swab, rectal swab, and clinical samples, respectively. Among the CP critical GNB that carried carbapenemase-encoding genes confirmed by PCR, the most common isolates was *K*.*pneumoniae* followed by *Acinetobacter*spp., *E*. *coli* and *P*. *aeruginosa*. This finding is in line with reports from China that *K*. *pneumoniae*, *E*. *coli*, *A*.*baumannii*, and *P*. *aeruginosa* were the predominant isolates CP-GNB [13]. Although the frequency varied, CP critical GNB carrying carbapenemase-encoding genes, particularly *K. pneumoniae* and *Acinetobacter* spp., were detected across all sample sources, including clinical, rectal, and environmental samples. The presence of CP critical GNB carrying carbapenemase-encoding genes in all sources sample types may indicate the circulation of these isolates between the environment and patients. Further advanced studies focusing on the transmission dynamics of CP critical GNB isolates are needed to clarify these transmission pathways and inform targeted infection control strategies.

This study showed that the prevalence of carbapenemase-encoding genes among carbapenem-resistant critical GNB was 78.7%. This is comparable to a previous study conducted in Ethiopia, which reported a prevalence of 69% [14], and a study from Sudan, where the prevalence was 58.75% [15]. However, it is higher than another study conducted in Ethiopia by Seman *et al*., which reported a prevalence of 44.5% [6]. The variation in prevalence rates may be due to differences in the number of carbapenemase-encoding genes targeted for detection, variations in the mechanisms of carbapenem resistance, and the types of GNB included in studies.

Among carbapenemase-encoding genes, the *bla*_NDM_ gene was predominant (75.9%) in CP critical GNB isolates. *bla*_NDM_ was first reported in India, and since its initial detection, *bla*_NDM_ carrying organisms have disseminated globally [16]. In Ethiopia, the first report of *bla*_NDM_ presence was in three *A. baumannii isolates* [17]; however, subsequent studies have identified its occurrence in Enterobacteriaceae [7] and *P*. *aeruginosa* [14,18]. In this study, the *bla*_NDM_ gene was found across all CP critical GNB species, including *K. pneumoniae, E. coli, Acinetobacter* spp., and *P. aeruginosa*, indicating its ease of circulation within and between bacterial species. This may also explain *bla*_NDM_ genes are encoded on highly mobile, conjugative plasmids which ease horizontal inter and intra-species transfer between bacteria [19,20]. Our results are consistent with previous studies conducted in Ethiopia [7,14] and other countries, which also reported the dominance of *bla*_NDM_ genes among GNB isolates [16]. The occurrence of NDM producing GNB in Ethiopia may be explained by several factors, including frequent travel between Ethiopia and NDM-endemic countries, due to weak infection prevention and control practices in healthcare settings, clonal expansion of NDM producing isolates, and/or the efficient spread of conjugative plasmids carrying the *bla*_NDM_ gene [19,20].

Interestingly, this study identified isolates co-harboring multiple carbapenemase-encoding genes, including *bla*_NDM_ in combination with *bla*_OXA-23_, *bla*_OXA-48_, *bla*_OXA-58_, and *bla*_VIM_. Similar findings have been reported in other countries, where isolates carrying multiple carbapenemase-encoding genes have been detected [13,15,21,22]. *K. pneumoniae* and *Acinetobacter*spp. showed two or more carbapenemase-encoding genes as it was reported previously in other reports that, *K. pneumoniae* and *Acinetobacter* spp. have co-existence of multiple carbapenemase-encoding genes [14]. This co-existence of carbapenemase-encoding genes poses a significant therapeutic challenge due to limited treatment options and the potential for horizontal gene transfer, facilitating global dissemination.

The low prevalence of *bla*_VIM_, *bla*_OXA-23_, *bla*_OXA-58_ and *bla*_OXA-48_ carbapenemase-encoding genes among critical GNB was reported in this study. In contrast to global reports of a high prevalence of *bla*_KPC_ genes, we have not detected *bla*_KPC_ and *bla*_IMP_ genes among the tested isolates.This was in line with the previous studies done at Addis Ababa hospitals [6,23] and reports from Sudan [15]. Despite the first occurrence of a KPC producer dated from 1996 in the United States, nowadays, Mediterranean countries, especially Italy, Greece and Israel, are endemic for the *bla*_KPC_ gene [24]. The movement of people between Ethiopia and endemic countries, due to medical visitation, trade, employment, and unrestricted cross-border travel, may contribute to the dissemination of various carbapenemase variants within the country. Additionally, variations in the types and prevalence of carbapenemase-encoding genes among CP-GNB in different studies may be influenced by factors such as geographic region, study population, and the genetic structure of isolates [24,25].

The detection of CP *K*.*pneumoniae*, *Acinetobacter* spp., *E*. *coli*, and *P*. *aeruginosa* on various ICU environmental surfaces underscores the significant role of the hospital environment in harboring and potentially transmitting antimicrobial-resistant pathogens. Notably, NDM-type carbapenemases were predominantly identified, particularly in *K*.*pneumoniae* and *Acinetobacter*spp.. This finding aligns with previous research indicating the widespread presence of NDM variants in healthcare settings [26].The co-existence of multiple carbapenemase encoding genes, such as *bla*_NDM_ and *bla*_OXA-23_ genes, within *Acinetobacter* spp. suggests a high potential for horizontal gene transfer, thereby increasing the risk of MDR outbreaks [27]. The contamination of high-contact surfaces, including baby incubators, bed rails, and sinks, further emphasizes the need for stringent infection control measures, as these areas may serve as reservoirs for CP critical GNB [26]. Moreover, these findings highlight the critical importance of routine environmental screening and the implementation of targeted disinfection strategies to mitigate the spread of resistant pathogens within ICU healthcare settings.

The frequency of CP critical GNB isolates with different carbapenemase-encoding genes across sample sources was varied. *K. pneumoniae* was found in both GI colonization and environmental samples, with a higher presence in GI colonization. This indicates that asymptomatic carriers and contaminated surfaces may serve as reservoirs, contributing to the spread and eventually increase in infection cases [9,28]. In contrast, *Acinetobacter* spp. was more frequently detected in environmental samples rather than in GI colonization. Likewise, the presence of multiple carbapenemase-encoding gene combinations, such as *bla*_*NDM*_ + *bla*_*OXA-23*_ and *bla*_*NDM*_ + *bla*_*VIM*_ was observed in *Acinetobacter* spp. from both infection and environmental samples. This reinforces the possibility that patients may acquire these resistant strains from environmental surfaces during the healthcare process [29,30]. For *E. coli*, the majority of carbapenemase-positive isolates were identified in GI colonization samples, with fewer found in environmental samples. This may support a study suggesting that *E. coli* primarily remains a gut colonizer, with a lower likelihood of transmission to the hospital environment or progression to infection [28]. *P. aeruginosa* with carbapenemase-encoding gene, particularly *bla*_*NDM*_*,* was detected in a sink, supporting its ability to persist in moist environments and contribute to outbreaks in critically ill patients. The presence of *bla*_*VIM*_ in an infection isolate further highlights the ability of *P. aeruginosa* to acquire and express carbapenemase genes, posing significant challenges in treatment and infection control [31,32].

One of the limitations of this study is that the environmental sampling was conducted after the COVID-19 pandemic, during a period when infection prevention and control (IPC) measures may have been intensified. This could have influenced the detection rate of critical GNB in the environment, underestimating the true level of contamination.

## Conclusions

This study demonstrates the presence of carbapenemase-encoding genes among critical GNB in ICU environmental contamination, GI colonization, and infections. The detection of critical GNB carrying these genes across all sample sources suggests possible circulation of these isolates between the environment and patients, highlighting the need for further research on transmission dynamics. Moreover, the identification of isolates harboring multiple carbapenemase genes underscores the importance of strengthening infection control measures in ICU settings.

## Supporting information

S1 Fig Agarose gel electrophoresis (1.5% agarose, run for 50 min). A. *bla*_NDM_, *bla*_KPC_ and *bla*_OXA-48_, Lane PC1,PC2 and PC3:positive control for *bla*_NDM_, *bla*_KPC_ and *bla*_OXA-48_ respectively, B. *bla*_OXA-58_ and *bla*_OXA-23_, Lane PC1 and PC2: positive control for *bla*_OXA-23_ and *bla*_OXA-58_ respectively C. *bla*_VIM_ and *bla*_IMP_ Lane PC1: positive control for *bla*_VIM_. First line Ladder (1,00 bp), NC: negative control, Lanes 1,2,3…: represent of isolates bands.
(TIF)

S1 Table Row data for carbapenem resistance critical GNB obtained from ICU patients with GI colonization and/orinfection, as well as from ICU environmental samples in the Amhara National Regional state, Ethiopia, 2021 to 2022 (XLSX)

S2 Table Primer sequences used for the detection carbapenemase-encoding genes. For degenerate primers: D = A, G or T; R = A or G; Y = C or T; K = G or T; W = A or T.
(DOCX)

S1 Raw Images The original uncropped and unadjusted images underlying all blot or gel results reported in S1 Fig.
(PDF)
