# Supplementary figures and images for: Carbapenemase-encoding genes in critical gram-negative bacteria isolated from ICU patients with infections and/or gastrointestinal carriage, and environmental samples in the amhara National Regional state, Ethiopia
Source: PLoS One. 2025 Sep 4;20(9):e0330613. doi: 10.1371/journal.pone.0330613 (PMC12410799; doi:10.1371/journal.pone.0330613)

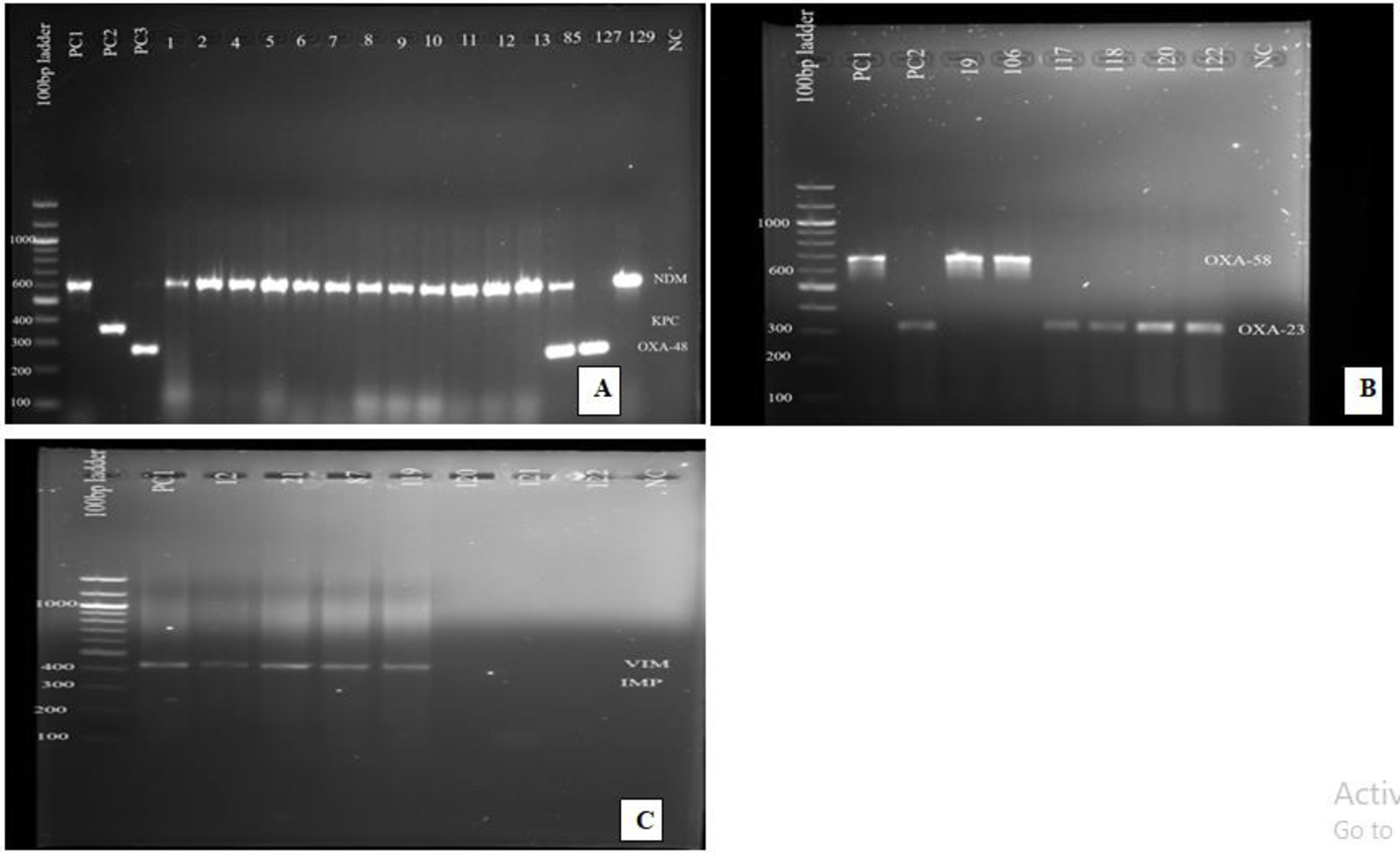

Supplement: S1 Fig — A. blaNDM, blaKPC and blaOXA-48, Lane PC1,PC2 and PC3:positive control for blaNDM, blaKPC and blaOXA-48 respectively, B. blaOXA-58 and blaOXA-23, Lane PC1 and PC2: positive control for blaOXA-23 and blaOXA-58 respectively C. blaVIM and blaIMP Lane PC1: positive control for blaVIM. First line Ladder (1,00 bp), NC: negative control, Lanes 1,2,3…: represent of isolates bands. (TIF) [file pone.0330613.s001.tif]

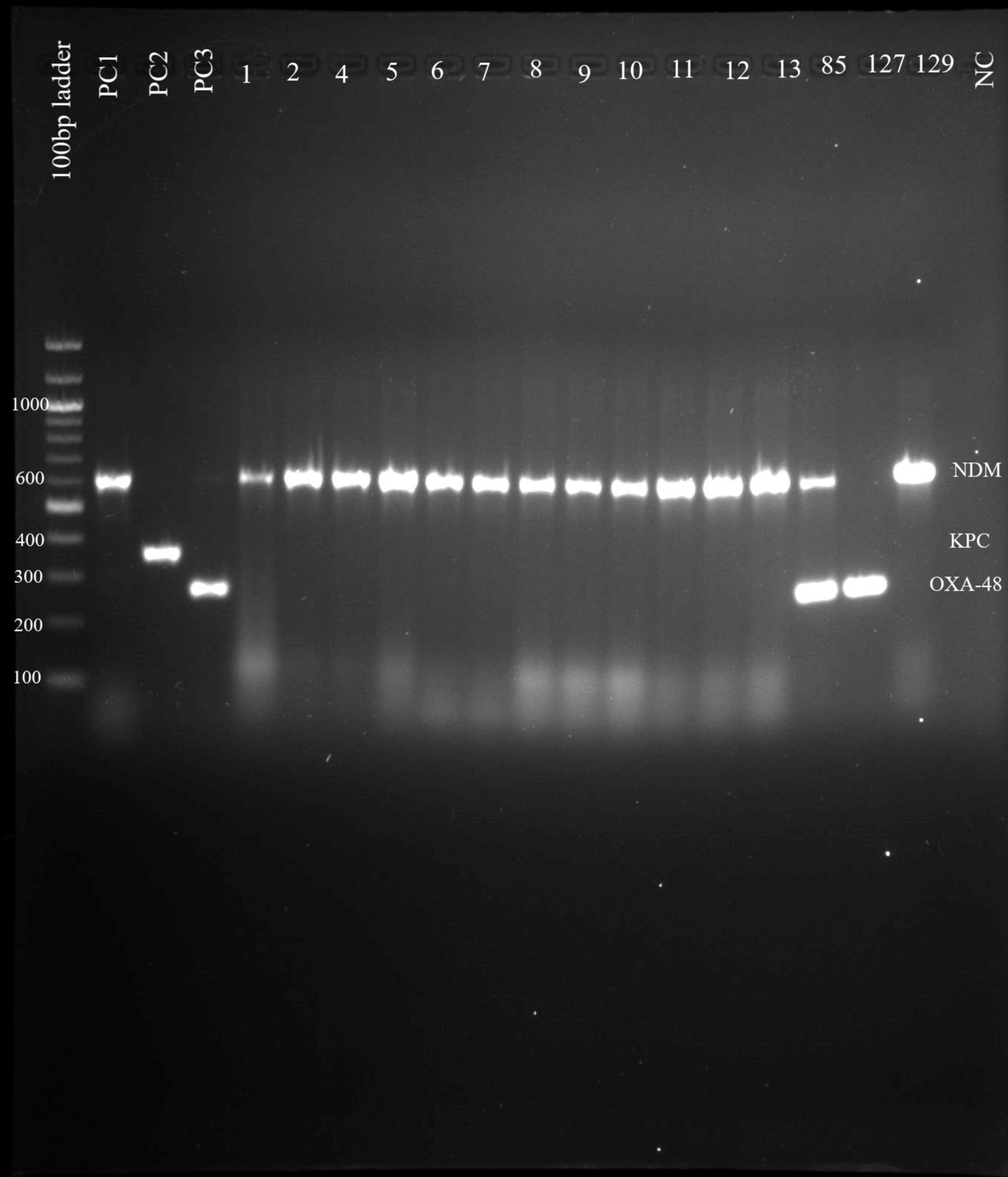

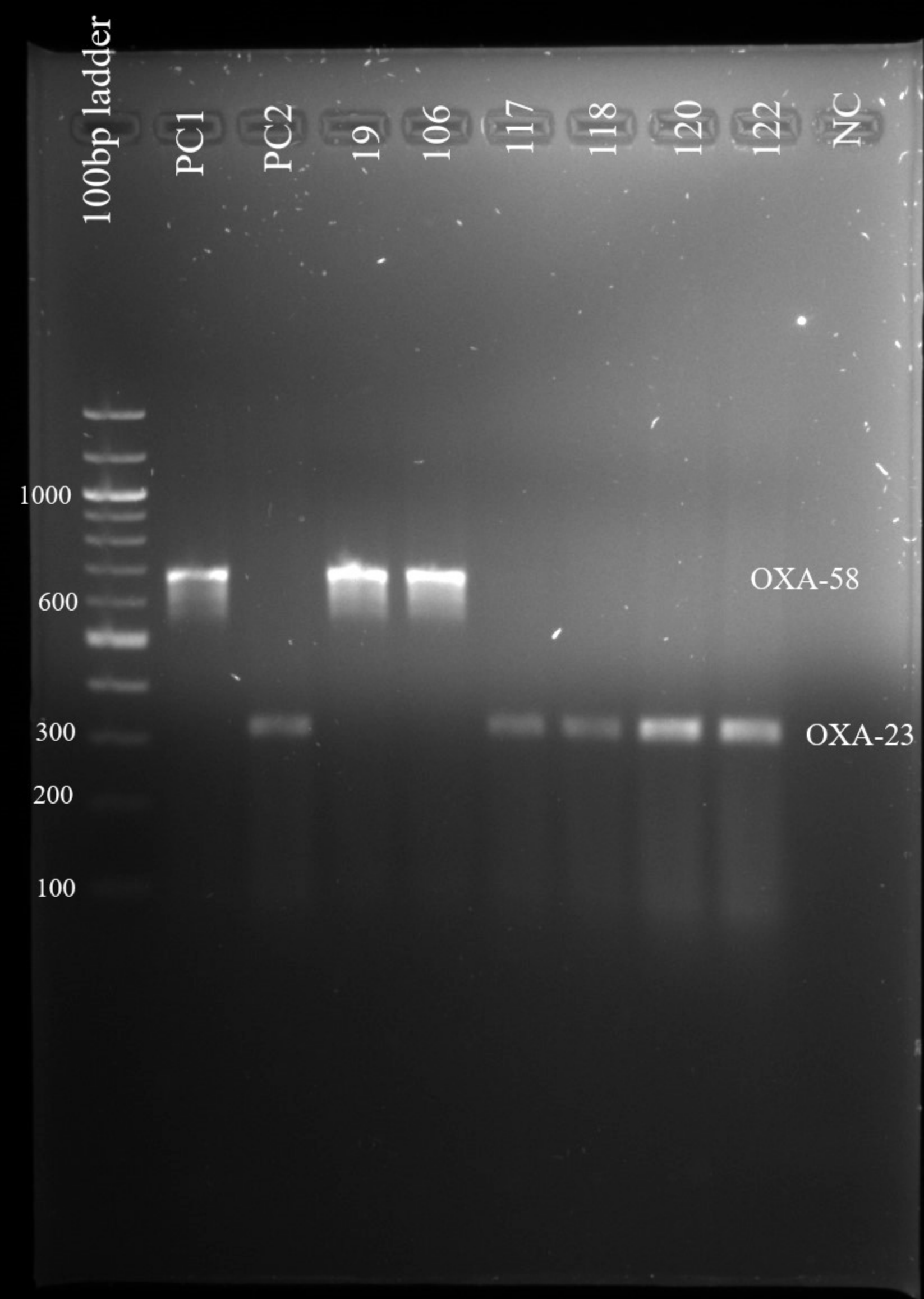

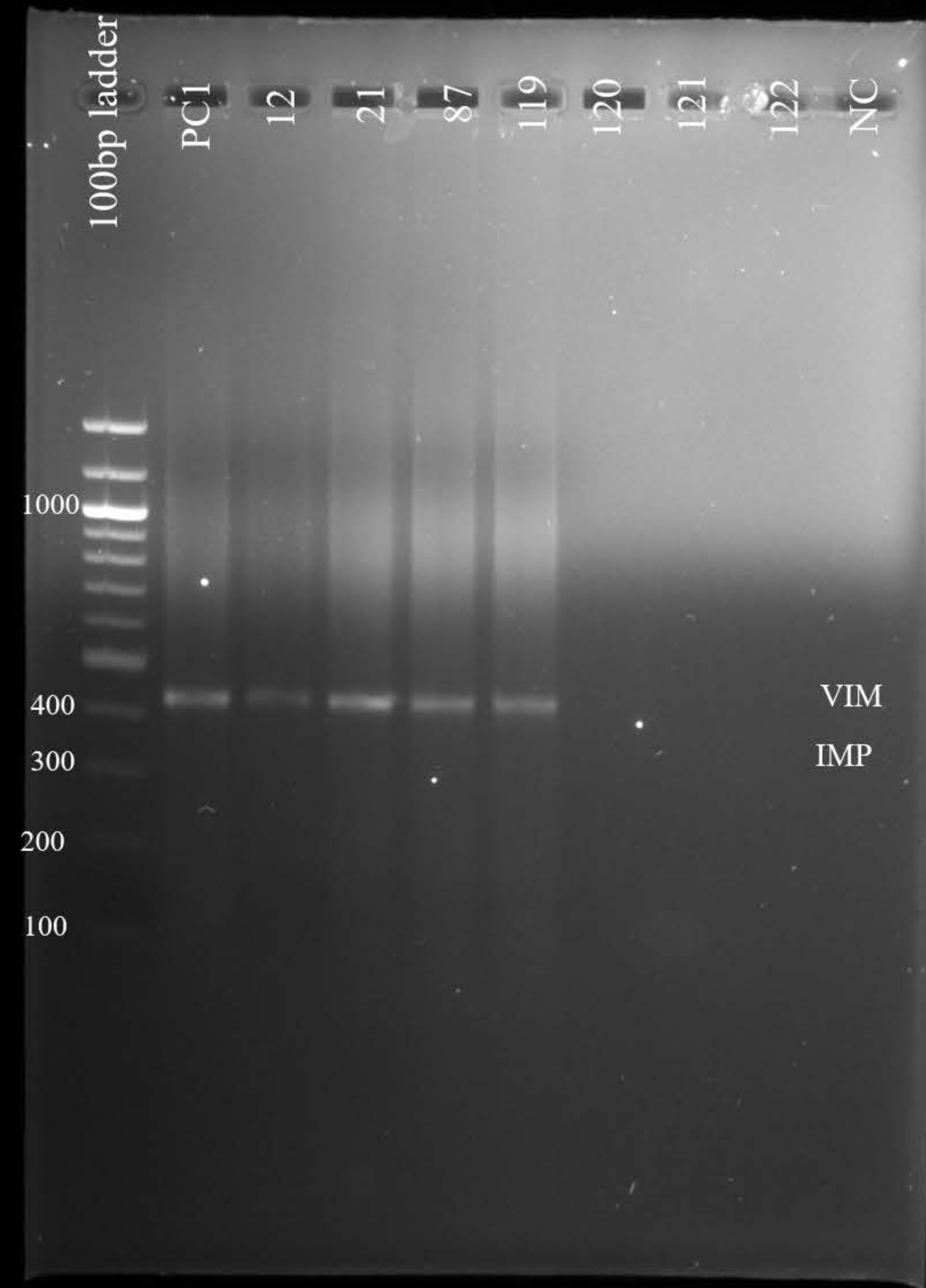

Supplement: S1 Raw Images — S1 Fig. (PDF) [file pone.0330613.s004.pdf]
