# Supplementary material for: Carbapenemase-encoding genes in critical gram-negative bacteria isolated from ICU patients with infections and/or gastrointestinal carriage, and environmental samples in the amhara National Regional state, Ethiopia
Source: PLoS One. 2025 Sep 4;20(9):e0330613. doi: 10.1371/journal.pone.0330613 (PMC12410799; doi:10.1371/journal.pone.0330613)
Supplement: S2 Table — For degenerate primers: D = A, G or T; R = A or G; Y = C or T; K = G or T; W = A or T. (DOCX) [file pone.0330613.s003.docx]

| PCR Name | Gene Targeted | Primer sequence(5’ to 3’) | | Amplicon size | References |
| --- | --- | --- | --- | --- | --- |
| Multiplex-1 | *bla*_NDM_ | F-primer | ACT TGG CCT TGC TGT CCT T | 603 | (11) |
|  |  | R- primer | CAT TAG CCG CTG CAT TGA T |  |  |
|  | *bla*_OXA-48_ | F-primer | ATG CGT GTA TTA GCC TTA TCG | 265 | “ |
|  |  | R- primer | CAT CCT TAA CCA CGC CCA AAT C |  |  |
|  | *bla*_KPC_ | F-primer | TCG CCG TCT AGT TCT GCT GTC TTG | 353 | “ |
|  |  | R- primer | ACA GCT CCG CCA CCG TCA T |  |  |
| Multiplex-2 | *bla*_OXA-23_ group | F-primer | CCC CGA GTC AGA TTG TTC AAG G | 330 | “ |
|  |  | R- primer | TAC GTC GCG CAA GTT CCT GA |  |  |
|  | *bla*_OXA-58_ group | F-primer | GGG GCT TGT GCT GAG CAT AGT | 688 | “ |
|  |  | R- primer | CCA CTT GCC CAT CTG CCT TT |  |  |
| Singleplex VIM | *bla*_VIM_ | F-primer | TGT CCG TGA TGG TGA TGA GT | 437 | “ |
|  |  | R- primer | ATT CAG CCA GAT CGG CAT C |  |  |
| Singeplex IMP | *bla*_IMP_ | F-primer | ACA YGG YTT RGT DGT KCT TG | 387 | “ |
|  |  | R- primer | GGT TTA AYA AAR CAA CCA CC |  |  |
